# Understanding the Drivers of Ghanaian Citizens' Adoption Intentions of Mobile Health Services

**DOI:** 10.3389/fpubh.2022.906106

**Published:** 2022-06-14

**Authors:** Isaac Kofi Mensah

**Affiliations:** Department of Business Administration, School of Economics and Management, Jiangxi University of Science and Technology, Ganzhou, China

**Keywords:** mobile health services, mobile technology, adoption intentions, Technology Acceptance Model (TAM), Ghana

## Abstract

Mobile health (m-health) application development and diffusion in developing countries have always been a challenge; therefore, research that seeks to provide an elucidation of the drivers of m-Health adoption is vital. Mobile health information systems and applications can contribute to the delivery of a good healthcare system. This study examined the factors influencing citizens' adoption of mobile health services. The Technology Acceptance Model (TAM) was used as the research underpinning for this study, while the data gathered were analyzed with SmartPLS through the use of the structural equation modeling technique. The results showed that perceived usefulness and ease of use were both significant predictors of the behavioral intention to use and recommend the adoption of mobile health services. Also, perceived risk was negative but significant in predicting the intention to use and recommend adoption. Mobile self-efficacy was found to significantly determine the behavioral intention to use, intention to recommend, perceived usefulness, and perceived ease of use of mobile health services. Besides, word-of-mouth showed a positive impact on both the intention to use and recommend. Contrary to expectations, the intention to use had no significant impact on the recommendation intention. The theoretical and practical implications of these findings are thoroughly examined.

## Introduction

Developing countries are taking advantage of the proliferation of mobile technologies and higher subscriptions of mobile phone usage to develop social and economic-related mobile applications to achieve a certain level of social and economic development. One critical social and economic application of mobile technology is in the context of healthcare delivery where mobile applications and mobile phones are used to provide quality healthcare services. Mobile technologies can improve communication and innovation in healthcare delivery (1, 2). The use of mobile technologies to deliver health care is known as mobile health (m-Health). It is defined as the delivery of health services and information through the utilization of mobile technologies like smartphones, 3G/4G/5G mobile networks, and satellite communications (3, 4). It is also considered as the use of the mobile phone's core utility of voice and short messaging service (SMS) among other applications such as general packet radio service (GPRS), global positioning system (GPS), generation mobile telecommunications (3G/4G/5G), and Bluetooth technology to deliver public health services (5). The development and deployment of m-health can ensure benefits such as remote monitoring, remote consultation, personal healthcare digital services, tracking of patients' health conditions, quick treatment, quality health data management, saving time and cost of diagnosis, and effective interaction with healthcare professionals (3, 6, 7). M-health has the potential to reduce or remove the geographical restrictions associated with access to quality healthcare provisions (8, 9).

The integration of mobile devices and their related technology in the health delivery system present an unparalleled chance to drastically transform the provision of health services to people, particularly in developing countries where the public health delivery system is dysfunctional (10). According to the World Health Organization (WHO), emerging sophisticated internet technologies and mobile networks coupled with high speed data transmission and cheaper and more powerful mobile devices are changing how health services and information are accessed, delivered, and managed (5). M-health is considered by most governments in low and middle-income countries as a complementary strategy to strengthen public health systems and attain the Millennium Development Goals (MDGs) health-related goals (5). Public health facilities have deployed m-health systems in areas such as maternal and child health, timely access to emergency, and general health services and information, reducing drug shortage in health centers and enhancing clinical diagnosis and treatment adherence (5). The WHO reports that the most frequently used m-health applications in countries globally are: health call centers/healthcare telephone lines (59%), emergency toll-free telephone services (55%), emergencies (54%), and mobile telemedicine (49%), and that the least reported mobile health initiatives include health surveys (26%), surveillance (26%), awareness creation (23%), and decision support systems (19%) (5).

The objective of this study is to explore the adoption of m-health services in the context of a developing country in West Africa, Ghana. Developing countries like Ghana can take advantage of innovations in mobile health technology to provide essential medical healthcare services to residents in rural and underdeveloped regions. The huge financial investment made in the development of mobile-driven health systems will amount to nothing if there is absence of adequate corresponding utilization by users. Adoption studies, thus, provide insights and fertile grounds to understand the drivers of mobile health utilization to provide policymakers with the needed ingredients for the development of m-health systems that meet the health expectations of the people, particularly in deprived regions, in a timely and cost- and resource-efficient manner. It has been emphasized that Ghana's healthcare infrastructure has undergone tremendous transformation; thus, it is imperative and crucial to measure the behavior of its citizens in a systematic way toward changes in the healthcare delivery systems (11). It has been articulated that m-health interventions go beyond the technology employed and are driven by individual and context-specific elements; thus, usability studies are needed to optimize the successful implementation of m-health systems (12). To attain the objective of this article, the Technology Acceptance Model (TAM) was integrated with new constructs such as perceived risk, mobile self-efficacy, word-of-mouth, and recommendation behavior of m-health systems along with the core variables of TAM like perceived usefulness and perceived ease of use. The new constructs integrated into the TAM contribute to the mobile health adoption literature especially when it comes to word-of-mouth communications and recommendations of m-health systems.

There are mobile health adoption studies in Ghana, but these studies (13–15) have not expanded on how factors such as perceived risk, mobile self-efficacy, and word-of-mouth communications can drive the uptake and recommendation of m-health systems among users and beyond. For instance, three dimensions of m-health service quality, such as system, interaction, and information quality, were found to drive the continual unitization of m-health services./system in Ghana (14), but this study (14) failed to explore how perceived risk, mobile self-efficacy, and word-of-mouth communications could influence user recommendations of m-health service. Coupled with this, there are limited studies that have undertaken research related to m-health adoption in Ghana. The research questions to be explored to address the gap identified are: what factors influence the adoption of m-health services among citizens, and what is the significant relationship among these factors? The conduct of this study will provide valuable input for policymakers to fashion and develop mobile health policies and systems to improve the uninterrupted delivery of quality health services to citizens in Ghana, especially in areas such as maternal health, prenatal care, infant care, HIV/AIDs prevention, treatment adherence, cardiovascular diseases, diabetes, and health promotion. The key findings and contributions of this study have shown that mobile self-efficacy determines the perceived ease of use and usefulness of mobile health services and both the behavioral intention to use and recommend the adoption of mobile health services. Besides, word-of-mouth communication (WOM) was found to influence the intention to use and recommendation intentions of mobile health services. These findings and contributions have enriched the m-health adoption literature from the perspective of Ghanaian citizens.

## Literature Review

### Mobile Health

The recent advancement in mobile computing technology, as well as mobile connections, has provided a basis for the development of mobile health (m-health) technologies to improve the standard of quality clinical healthcare worldwide (16, 17). Mobile technologies have great capacities to deliver a quality and healthy lifestyle for everyone due to their mobility power (18). Also, mobile technologies/devices are designed with sensors and unique characteristics that enable healthcare professionals to attend to patients with constant connectivity and can support patient-doctor interactions 24/7 at any period (19). M-health promotes the delivery of quality healthcare administration, which empowers healthcare professionals to collect clinical data, monitor the health status of patients, check information, and make prognoses for anticipated health challenges (19, 20). In addition to improving and facilitating the delivery of healthcare, m-health has integrated functions such as medical prescription electronically, enabled clinical decision making, self-care and code scanning, billing services, and e-learning functions (19, 21). M-health is intended to achieve improved health outcomes, healthcare quality, and health equity through the use of mobile systems for medical data, track and get feedback on medications and appointments, and empower online visits with physicians (22, 23). Decentralized personal health records can be achieved better through mobile systems to empower patients to access their medical records in a secured and confidential manner without interruption (24).

While the development and deployment of mobile health services/systems are crucial to providing quality healthcare to citizens, the equally essential question that needs to be considered is the factors influencing citizens' decision to use mobile health services. A broader understanding of the factors attracting citizens' adoption intentions of mobile health services would provide quality information to the government, public health practitioners, and policymakers to fashion and design mobile health policies, systems, and strategies to ensure maximum utilization and adoption. Several adoption studies, particularly in developing countries like Bangladesh, have shown that effort expectancy, performance expectancy, social influence, facilitation conditions, technology anxiety, and resistance to change were all positively related to the adoption of mobile health services among the elderly in Bangladesh (25). Among citizens of Harbin, China, it was demonstrated that while subjective norms and perceived ease of use were found to determine the adoption of mobile health services, perceived security was not significant in influencing the adoption intentions (26). These adoption studies help policymakers to develop m-health services and systems that will be widely adopted and accepted within a particular jurisdiction, since m-health service systems are designed based on cultural and unique characteristics of a country (27).

A study on mobile health application utilization during the coronavirus disease 2019 (COVID-19) pandemic showed that the decision of users to adopt mobile applications for COVID-19 surveillance was influenced by perceived usefulness, perceived ease of use, and event-associated fear (28). The study also indicated that women showed more anxiety disorders than men in the utilization of mobile health apps (28). In assessing the mobile health services for the elderly in Pakistan, it was demonstrated that performance expectancy, effort expectancy, social influence, facilitating conditions, perceived ubiquity, and perceived trust drive m-health behavioral adoption (29). A similar study conducted in China showed that performance expectancy, effort expectancy, and facilitating conditions influence the readiness of citizens to use mobile health systems (30). In Malaysia, it was determined that for mobile health systems to adopt issues of relative advantage, perceived ease of use, compatibility, observability, trialability, and perceived risk should be considered in the development of m-health systems (31).

### Mobile Health Applications

A critical component of mobile health development and integration in the healthcare administration system is the development of mobile health applications (apps) that can be used to promote the delivery of mobile health. These m-health applications are designed for the learning, treatment, diagnostic, and accomplishment of certain health goals (19). M-health apps are also designed to ensure the treatment of psychological diseases through constant interaction between patients and their healthcare professionals (19). Most m-health applications are built on Android and IOS operating systems, and they fall under two areas, i.e., apps used by healthcare professionals and apps used by patients. The apps used by healthcare professionals can be classified as literature (health), patient monitoring and diagnosis, personal care applications, psychological health applications, educational applications, or social networking applications (19). Patients' m-health apps include care applications (such as fitness, sports, games, and auto diagnosis), an app to find out about PHR, apps to contact doctors/healthcare professionals, educational health applications, and social networking applications (19). Android smartphone sensors have been used to develop context-aware ambient intelligent applications for healthcare monitoring and delivery because of their easy-to-carry characteristic (32). Android smartphone sensors are considered to generate precise, reliable, and more accurate results as far as physical activity recognition is concerned compared with other approaches such as body sensors (32). The trend is moving toward smart sensors because of their valuable, precise, and accurate nature (33).

M-health applications can be considered as the driving force for the success of the mobile health concept. The classifications of m-health applications and their characteristics are outlined in Table 1. Table 1 was adapted from Pires and Marques (19).

**Table 1:** M-health application classifications and characteristics.

**Mobile applications**	**System**	**Characteristics/** **Description**
**Classification 1: M-health apps regarding literature**		
Speed muscles MD (muscular dystrophy)	Android and iOS (iPhone Operating System)	Anatomy study, the speed, and memory of identifying the muscle tests
Speed Angiology MD (muscular dystrophy)		Examines anatomy, check speed and memory of knowing the arteries and veins
Medscape		Presents many drug references, diseases library, procedures, and protocols
Quick LabRef	Android	Offers a faster latest information on the recent widely used clinical laboratory values
WomanLog Calendar	Android and iOS(iPhone Operating System)	Indicates a menstrual and fertility calendar for women empowers the women to be aware of their fertile section.
**Classification 2: M-health applications regarding diagnosis and treatment**		
iTriage	Android	Determines the health conditions of the patient; locates a health care provisional within their location
Diabetes Buddy	iOS (iPhone Operating System)	Patients can manage diabetes, tract factors that cause blood sugar levels, monitor the fluctuations of the blood sugar level, data sharing with health professionals
Glucose Buddy	Android and iOS (iPhone Operating System)	Monitors glucose levels, food consumption, insulin dosage, permits sending information gathered by email
**Classification 3:M-health applications concerning personal care applications**		
Cook IT Allergy Free	Android	Library of recipes for those sensitive to gluten, dairy, eggs, nuts, provides substitutions and customization of recipes
MyPlate	Android and iOS (iPhone Operating System)	Control the user's diet, weight change, and workout to keep fit
Mindful Eating		Builds alerts for people to watch what they eat gives badges for nutritional milestones and advises on good patterns.
**Classification 4: M-health applications for psychological goals**		
Awareness	iOS (iPhone Operating System)	Intercepts users' daily routines, prompts routines to get in touch with what they feel, provides insight, and breaks patterns of emotions, attitudes, and behavior through awareness and inspirational practices
Yoga Relax	Android	Information about poses, steps for correct positioning, and how to maintain a pose.
**Classification 5: M-health applications regarding educational and social networking applications**		
First Aid	Android and iOS (iPhone Operating System)	Provides information on urgent and emergent medical cases
draw MD(muscular dystrophy)-Patient Education	iOS (iPhone Operating System)	Enables healthcare professionals to draw out surgical procedures for their patients in an easy manner.
Doximity and DocBook MD (muscular dystrophy)	Android and iOS (iPhone Operating System)	Enables health professionals to find others wanting to communicate and improves communication among health professionals
Univadis US		Empower health care professionals to learn and improve their practices and have access to forums in medical research, clinical care, policy, and regulations.

### Mobile Health in Ghana

Since 2004, Ghana has initiated, deployed, and piloted about 22 mobile health projects to improve health delivery systems in the country (5, 34, 35). Ghana as a developing country has, thus, made attempts to integrate mobile technologies in the health delivery system to ensure a reliable system of communication for consultation and referral of patients. In 2008, the Ghana Medical Association (GMA), with support from donor partners, deployed the Mobile Doctors Network (MDNet), which was called the Medicare Line Program (5). It was the first mobile health application deployment that provided free mobile-to-mobile voice and text services to physicians in Ghana (5). It also facilitated the dissemination of information to doctors concerning national emergencies and meetings and interactions with doctors (5). The lack of access to computers and low penetration of internet services particularly in rural parts of the country prompted the use of cellular phones as an alternative to achieve efficient and culturally responsive healthcare (5). The development of the MDNet program contributed to reducing the cost barrier for clinical consultations among doctors and, thus, improving the provision of medical advice and referral of patients who require special medical care to other centers in Ghana (5). Importantly, the MDNet improved communication about patient management among physicians in the country's health delivery system, and through this system, complicated medical issues were managed like getting information about specialist doctors, bed availability, and clinic periods/times and referrals (5).

Another important mobile health project initiated in Ghana is known as the Mobile Technology for Community Health (MOTECH), which was launched in August 2010. The ultimate goal of this project was to improve the quality (wellbeing) of women during pregnancy and postpartum (15). The MOTECH project was developed to empower and enable pregnant women and new mothers to improve knowledge and understanding of health services *via* mobile phone-mediated voice messages on maternal, newborn, and child health and the efficient management of appointments and notifications for emergency healthcare services (15). The MOTECH program also facilitates the collection of patient-level clinical information, improves data-reporting processes, and enhances prompt delivery of maternal, newborn, and child health (MNCH) services. MOTECH was made up of two parts: Mobile Midwife and Client Data Application (CDA). Mobile Midwife was intended to improve patients' knowledge and awareness of important health information during the period of pregnancy, while CDA provided health workers with the opportunity to use mobile devices to collect information for better track and provide quality and timely delivery of health services to pregnant women (15).

### Recent Mobile Health Studies in Ghana

Ghana, with a population of about 30 million citizens, is taking advantage of the proliferation of mobile technology, mobile handsets, and wireless networks to improve the access and delivery of public health. Ghana is one of the first nations in Africa to have access to a mobile cellular network and has since developed a robust infrastructure (36) to support the continued use of mobile technology to drive the economic and social development of the country. In Ghana, it has been illustrated that access to mobile phones improves the well-being of households in terms of being “non-poor” (37). Investment in the right ICT infrastructure to increase access, penetration, and quality of service especially in undeveloped regions (37) can serve as the foundation for the development of mobile-enabled services such as mobile health services.

M-health interventions were seen and continue to be seen as acceptable and practicable to address the challenges confronting the delivery of quality health services particularly in deprived areas of Ghana (38). Some m-health interventions or applications studies in Ghana have indicated that m-health technology can be used to improve maternal and child health services (38). It was further elaborated that m-health interventions reduce the barriers to equitable access to maternal and child care services by women in rural settings (38). Knowledge and awareness of m-health intervention were high among citizens, and there were active positive attitudes toward the use of mobile phones to receive health care (38, 39). Another critical sector of the intervention of m-health is mental healthcare. An innovative m-health infrastructure can be used to overcome deficiencies in the public healthcare delivery system (36). Major stakeholders such as patients, providers, government officials, and traditional/faith practitioners welcome the adoption of m-health technologies to promote adequate and humane care, reduce human rights violations, and enhance clinical outcomes (36).

### Barriers to m-Health in Ghana

Ghana, like any country that harnesses mobile technology to provide m-health services, is also confronted with many challenges in the development and deployment of mobile health technology and services. Limited or erratic power supply and poor mobile network connectivity have been identified as some of the barriers to the implementation of m-health in Ghana (38). The barriers to mobile technology integration into the health administration system include infrastructure, usability, acceptability, integration of technology and interoperability, data security and privacy, reliability and network access, unstrained handler/staff/personnel, illiteracy, financial accessibility, and policy and regulations (40).

#### Infrastructure

Deficit in infrastructure development has always been a major setback for the development and implementation of mobile-led applications such as m-health applications. Particularly for developing countries like Ghana where over the years the amount of financial investment in the mobile technology infrastructure base has been neglected is a key challenge in the development of m-health system. Technological infrastructures such as Wi-Fi, Bluetooth, and cellular data connection are vital to influence the quality of m-health development (41).

#### Technology Integration and Interoperability

The integration of technology along with its interoperability is vital for the success of mobile-driven apps to have access to other systems. The capacity of m-health systems to update, merge, and be used across different technological systems has been a challenge, since it can impact negatively on the level of uptake because of its inability to work on other technological systems (40, 42).

#### Data Security and Privacy

Security and privacy issues have often been the major concerns for users of any technological system, and that applies to mobile health technology as well. The data provided by a patient are vital for clinical treatment; thus, any form of breach or disclosure to a third party may cause severe trauma because of the public ridicule that the patient may confronted (40, 43). It has been indicated that m-health patient data confidentiality has often been an issue, since data capture, storage, and retrieval processes are not managed effectively (43).

#### Network Access and Reliability

Mobile health technology is dependent on the level of availability and affordability of network access, speed, and signal strength (40). Bad connection and signals in certain geographical locations can affect access to health through mobile health systems. It has been elaborated that for m-health to be beneficial to targeted users, especially in deprived regions, adequate universal network coverage should be a top priority (44).

####  Illiteracy

The issue of illiteracy has often been a concern in the introduction of new technological systems like mobile health systems. The lack of education renders some users unable to comprehend and manipulate the system to their benefit, and this may affect the functionality of the device to non-regular updates (40, 45). The absence of literacy among users affects the potential of users to locate, evaluate, and effectively use information technological systems (45).

#### Policy and Regulation

Government policies and regulations are instrumental in driving the concept, development, and implementation of mobile health systems. The absence of adequate policies and regulations will be detrimental to the success of m-health adoption. Public policies and regulations are missing for the use of mobile health systems as an intervention for many countries including Ghana because of lack of adequate consultations among key stakeholders such as government policymakers, vendors, designers, health physicians, and users (40).

#### Financial Accessibility

The ability of users to afford the cost of technological devices needed to be able to have access to mobile health is a challenge for users in deprived areas in Ghana. This is echoed in (40), which indicated that the cost of apps and devices is a problem for the successful deployment of the m-health system. Also, the increasing cost of technical devices, coupled with high standard of living, reduces the desire of users to pay for m-health services (40). It has been suggested that improving the standard of living and stabilization of prices in deprived regions should be the goal of government and policymakers (46).

## Development Of Research Framework And Research Hypotheses

### Research Framework

#### Technology Adoption Theories/Models

Technology acceptance theories/models have been proposed and validated to examine the nature of information technology acceptance. Some of the popular adoption theories/models are The Unified Theory of Acceptance and Use of Technology (UTAUT) (47), the Technology Acceptance Model (TAM) (48), the Motivational Model (49), The Theory of Reasoned Action (TRA) (50), The Theory of Planned Behavior (TPB) (51), the Model of PC Utilization (52), Social Cognitive Theory (53), and Innovation Diffusion Theory (54). The Technology Acceptance Model (TAM) will be adapted as the theoretical framework for this study. However, descriptions of other models/theories, along with their core constructs, are shown in Table 2.

**Table 2:** Technology adoption theories/models description with key constructs.

**Technology adoption** **theories/models**	**Description with key constructs**
Theory of Reasoned Action (TRA)	TRA assumes that people's intention drives their actual behavior while the intention to use is predicted by attitude toward the use and subjective norms concerning the performance of such behavior.
The Unified Theory of Acceptance and Use of Technology (UTAUT)	UTAUT posits that the intention to adopt information technology is driven by key constructs such as performance expectancy, effort expectancy, and social influence. Facilitating conditions are presumed to affect the actual usage behavior. These factors' relationships are moderated by gender, age, experience, and voluntariness of use
The Social Cognitive Theory (SCT)	This theory suggests that environmental factors, personal factors, and behaviors are predicted reciprocally. This is opposed to TPB, TAM, and IDT assuming that there is only unidirectional causation among constructs in their models. SCT gives prominence to the self-efficacy concept.
The Innovation Diffusion Theory (IDT)	This model assumes that five attributes of innovation such as relative advantage, complexity, compatibility, trialability, and observability determine the acceptance and adoption behavior. This was expanded to include an image, visibility, results demonstrability, and voluntariness of use.
The Model of PC Utilization	Constructs such as job fit, complexity, long-term consequence, affect toward use, social factors and facilitation conditions are the assumptions that underline the PC utilization behavior.
The Motivation Model	This model utilizes the concept of motivational theory to study the adoption and use of information technology which is based on two core ideas: extrinsic and intrinsic motivations. Extrinsic motivation is the perception that users want to undertake an action because it can achieve outcomes that are distinct from the action itself. Intrinsic motivation is about the perceptions of pleasure and satisfaction obtained from undertaking a behavior.
Theory of Planned Behavior (TPB)	TPB is similar to TRA with TPB also assuming that individuals are rational decision-makers. The difference between TRA and TPB is that the former is used to predict people's behavior in a voluntary situation while the latter is for determining behavior in a mandatory context. A new construct of perceived behavioral control is added in TPB but holds the same TRA constructs.

#### Technology Acceptance Model

The TAM is one of the major technology adoption theories developed to explain the use of information technology or systems (48). Theories seeking to predict the decision of users to accept or reject a technology is grounded in the domain of psychology (55, 56). The explanation for the adoption and utilization of new forms of technology is premised on people's internal beliefs, attitudes, and intentions (57, 58). The TAM originated from the Theory of Reason Action (TRA) (59) and the Theory of Planned Behavior (TPB) (51). According to this model, the adoption of information technology is based on two main constructs, perceived usefulness and perceived ease of use (48). Attitude toward use, behavioral intention to use, and actual use of technology are some of the additional constructs in the TAM (48). In the TAM, both perceived usefulness and perceived ease of use influence individual attitudes toward the adoption of information technology as well as the intention and actual use of information systems (48). Also, perceived ease of use affects the perceived usefulness of the information technology system, and perceived ease of use and perceived usefulness are influenced by external constructs (48). Although the TAM has been revised (ETAM2) to take care of additional constructs such as attitude toward use, experience, job relevance, output quality, and subjective norm, the main concept of the model was undiluted (47, 57).

The Extended TAM (ETAM2) (60) was devised to identify external factors that drive the perceived usefulness (PU) of a technology. The extended constructs of PU are subjective norm, image, job relevance, output quality result demonstrability, and addition of experience and voluntariness as the moderating elements of the subjective norm (60). The subjective norm describes the influence of others on people's desire to use or not to utilize a technology (60, 61). Image has to do with the desire of people to maintain a good reputation or standing with others (55). Job relevance is considered the extent to which a technology is appropriate (55). The factor of output quality explains the degree to which a technology can sufficiently undertake the needed works/tasks and for that of the result demonstrability, is concerned with the production of tangible outcomes (55).

The potential of the TAM to provide a better explanation for user adoption of information system-related technology has been validated across many technology innovation applications such as e-health/mobile health (3, 62), e-commerce/mobile commerce (63–66), e-government/mobile government (67–69), and e-learning (70–72). These broader applications and validations of the TAM in these many fields of research provide the empirical basis for scholars and researchers to continue to rely on the constructs of TAM to understand the factors influencing the individual user perceptions toward the use of new technology related innovations. The numerous validations of the TAM along with its extensions do not only offer new perspectives on technology adoption but also importantly establish the TAM as a robust and relevant model in this current dispensation. Additionally, the results obtained from the utilization of the TAM are usually accepted as being actual and accurate measures of adoption and usage (57, 73). The TAM also has some inherent advantages as compared to other competing models: first, the TAM is direct and specific in the utilization of information systems in the context of usefulness and ease of use, second is that it is parsimonious, and third is that it is considered more robust in information system research and applications (74, 75). Thus, the TAM has considerably become the most influential theory (55). This empowered its application (TAM) in this study, since it can enable the adequacy and reliability of the outcomes of the m-health parameters examined in this study, which is based on the spirit and fundamentals of TAM.

A summary of TAM's applications (extended or modified) in recent information system research is shown in Table 3. TAM modifications have occurred as a result of the need to enhance by integration of supplementary constructs to validate the adequacy of resolving the question of technology adoption drivers from multiple dimensions. These studies demonstrate the extent to which the factors that influence the acceptance or rejection of technology can be a hindrance or positive to the process of knowledge transfer and acquisition empowered by technology (55, 82).

**Table 3:** TAM's recent application (extended/modified).

**References**	**Key results**	**Extended/Modified constructs**
Pal and Patra (76)	Using the combined TAM and Task-Technology-Fit Model to understand video-based learning during the COVI-19 pandemic, it was shown that perceived ease of use (PEOU) influences perceived usefulness (PU) and attitude, and PU influenced attitudes and actual usage. Also, technology characteristics (TC) and individual characteristics(IC) influenced task-technology fit (TTF), and TTF impacted PU and PEOU.	Task-Technology-Fit, Technology characteristics, individual characteristics, gender, inequality
Lu and Deng (77)	Using an extended TAM it was demonstrated that the acceptance of intelligent surveillance systems is driven by job relevance, government action, training, and technical support. PU, PEOU, and cost savings positively impacted the intention to use, perceived risk showed a negative impact on the intention to use.	Subjective norm, job relevance, top management support Government action, Training, Technical support, Technology Anxiety, cost savings, and privacy risk
Ishfaq and Mengxing (78)	Using TAM to explore the internet-based services during the peak of the COVID-19 it was revealed that FC impacts PEOU but not PU, SI does not impact both PEOU and PU, TTF drives PU but not PEOU, PU does not influence attitude, but PEOU does, and attitude influences intention to use	Facilitating conditions (FC), social influence (SI), task technology fit
An et al. (79)	Applying an extended TAM to understand the factors driving the adoption of telehealth after the flattening of the COVID-19 curve in South Korea, it was validated that increased accessibility, enhanced care, and ease of use of telehealth showed a positive impact on the perceived usefulness of telehealth. Also perceived usefulness, ease of use, and privacy/discomfort influence the acceptance of telehealth. The anxiety of COVID-19 was linked with the acceptance of telehealth.	Increased accessibility, enhanced care, privacy and discomfort, anxiety about COVID-19
Tsai et al. (80)	Using TAM to explore the deployment of masks to comeback the COVID-19 in Taiwan showed that the intention to use was predicted by attitude toward use, perceived ease of use, perceived usefulness, health literacy, and privacy and security.	Health privacy, privacy, security, and computer self-efficacy
Huarng et al. (81)	Understanding the adoption of healthcare wearable devices using TAM showed that the intention to use was determined by higher data privacy, perceived ease of use, and reliable data. However economic burden reduces the intention to adopt healthcare wearable devices	Economic burden, data privacy

### Hypothesis Development

#### Perceived Usefulness

Perceived usefulness is defined as the perception of individuals that the use of new information technology systems will contribute to improving their job performance (48). M-health services are technology-related; hence, its uses should bring added benefits as they seek quality healthcare services. In the context of mobile health perceived usefulness is the extent to which citizens understand that the use of mobile devices for healthcare services will bring some advantages to them (83). M-health services that provide a good environment for consumers to improve their access to quality healthcare will ultimately impact positively their perceptions of the usefulness of mobile health services. This means that users that feel m-health services are useful and will enhance their way of life and work performance and assist them in obtaining quality of life will encourage other people to use it. These positive perceptions of the usefulness of m-health services could have a corresponding impact on the behavioral intention of citizens to adopt m-health services. Previous studies have demonstrated that perceived usefulness is positively related to intention to use m-health services (3, 84–87). Benefits arising from the use of m-health services can also lead to their recommendation to others. Consequently, H1 and H2 were proposed.

H1: Perceived usefulness is positively related to the citizens' behavioral intention to adopt m-health services.
H2: Perceived usefulness is positively related to the citizens' intention to recommend the adoption of m-health services.

#### Perceived Ease of Use

The ease of use associated with the use of a particular technology is a critical factor that consumers or users will consider before its adoption. Perceived ease of use is defined as the extent to which individual users of technologies are convinced that the use of such technologies will be free of challenge or effort (48). Perceived ease of use, in the context of m-health, is the extent to which citizens consider that the use of m-health services to access healthcare will be free from any challenge or problems. Citizens' unimpeded access to health services through systems that are easy to use and, thus, provide a convenient environment for the adoption of mobile healthcare will drive them to use it. Ease of use features such as faster download time, easy to download and upload documents, interface customization, technical functionality, design, easy browsing, and navigation of mobile health service sites would enhance the perceived ease of using mobile health services. The comfort and convenience provided through the perceived ease of use of mobile health services can influence the citizens' behavioral intention to use. Studies have shown that perceived ease of use has a direct positive impact on behavioral intention to use (87–90). The perceived ease of use of mobile health services can also influence citizens' recommendation intentions. Accordingly, H3 and H4 were proposed.

H3: Perceived ease of use is positively related to the citizens' behavioral intention to adopt m-health services.
H4: Perceived ease of use is positively related to the citizens' intention to recommend the adoption of m-health services.

#### Perceived Risk

Perceived risk is considered one of the critical factors influencing the adoption of technology-related applications such as m-health services. Citizens may express concerns on the use of a mobile technology to deliver healthcare services because of risks that may arise from its use. The lack of a proper regulatory framework to protect users from the abuse and disclosure of their medical information and records to third parties without their prior approval may cause some level of fear or risk perceptions toward the adoption of mobile health services. Perceived risk, therefore, is the citizens' feeling or state of uncertainty or anxiety about the use of mobile health services. Privacy and security, performance, financial, social, and time risk concerns are some of the major risk considerations that consumers may consider and may play a role in reducing the level of confidence in the use of new technologies like m-health (91–93). Studies have shown that perceived risk is significantly and negatively related to behavioral intention to adopt (3, 88, 90). The extent of perceived risk can also determine the willingness of citizens to recommend the adoption of m-health services. Accordingly, H5 and H6 were proposed.

H5: Perceived risk is negatively related to the citizens' behavioral intention to adopt m-health services.
H6: Perceived risk is negatively related to the citizens' intention to recommend the adoption of m-health services.

#### Mobile Self-Efficacy

The diffusion and adoption of technology-related applications can be attributed to the level of self-efficacy that users possess. Self-efficacy is considered as the individual potential or belief in his or her ability to undertake and complete a particular course of action (94). Therefore, mobile self-efficacy can be defined as the confidence in the user's ability to use mobile devices to complete a particular course of action or exercise. Mobile self-efficacy, in the context of mobile health services, is the ability of citizens to independently use their mobile devices (mobile handsets) to successfully access public health services. Mobile self-efficacy has been demonstrated to have a direct impact on the adoption of technology relation systems. Specifically, studies have shown that mobile self-efficacy is significantly related to behavioral intention to use and both perceived usefulness and perceived ease of use (95–97). The level of citizens' mobile self-efficacy can also influence the intention to recommend the adoption of m-health services. H7, H8, H9, and H10 were proposed.

H7: Mobile self-efficacy is positively related to the citizens' behavioral intention to adopt m-health services.
H8: Mobile self-efficacy is positively related to the citizens' intention to recommend the adoption of m-health services.
H9: Mobile self-efficacy is positively related to the perceived usefulness of m-health services.
H10: Mobile self-efficacy is positively related to the perceived ease of use of m-health services.

#### Word of Mouth Communication

Word of mouth (WOM) communication is considered as one of the effective means for consumers to gather information about products and services to determine their adoption intentions or behavior decisions (98). WOM communication emanates from close friends and colleagues through their interaction about services or products. WOM communication is the expression of personal experiences (positive or negative) on products or services to people in their surroundings. In the context of mobile health services, WOM communication is defined as the sharing of citizens' perceptions or experiences concerning the use of mobile devices to provide health services and their attitudes toward adoption by friends, family, acquaintances, and other individuals around them. Studies have shown that WOM communication is positively related to perceived usefulness, perceived ease of use, and intention to use (99). WOM communication of m-health services can influence the citizens' adoption of m-health services and can lead to recommendation to others. Consequently, H11 and H12 were proposed.

H11: WOM communication is positively related to the citizens' behavioral intention to adopt m-health services.
H12: WOM communication is positively related to the citizens' intention to recommend the adoption of m-health services.

#### Behavioral Intention to Adopt

Behavioral intention to use a technology has the potential to influence users to likely adopt it and recommend its adoption to others as well (100, 101). Studies have shown that behavioral intention to use is positively related to the intention of users to recommend the adoption of technologies to others (102).

H13: Behavioral intention to adopt is positively related to the citizens' intention to recommend the adoption of m-health services.

## Research Model

**Figure 1:**
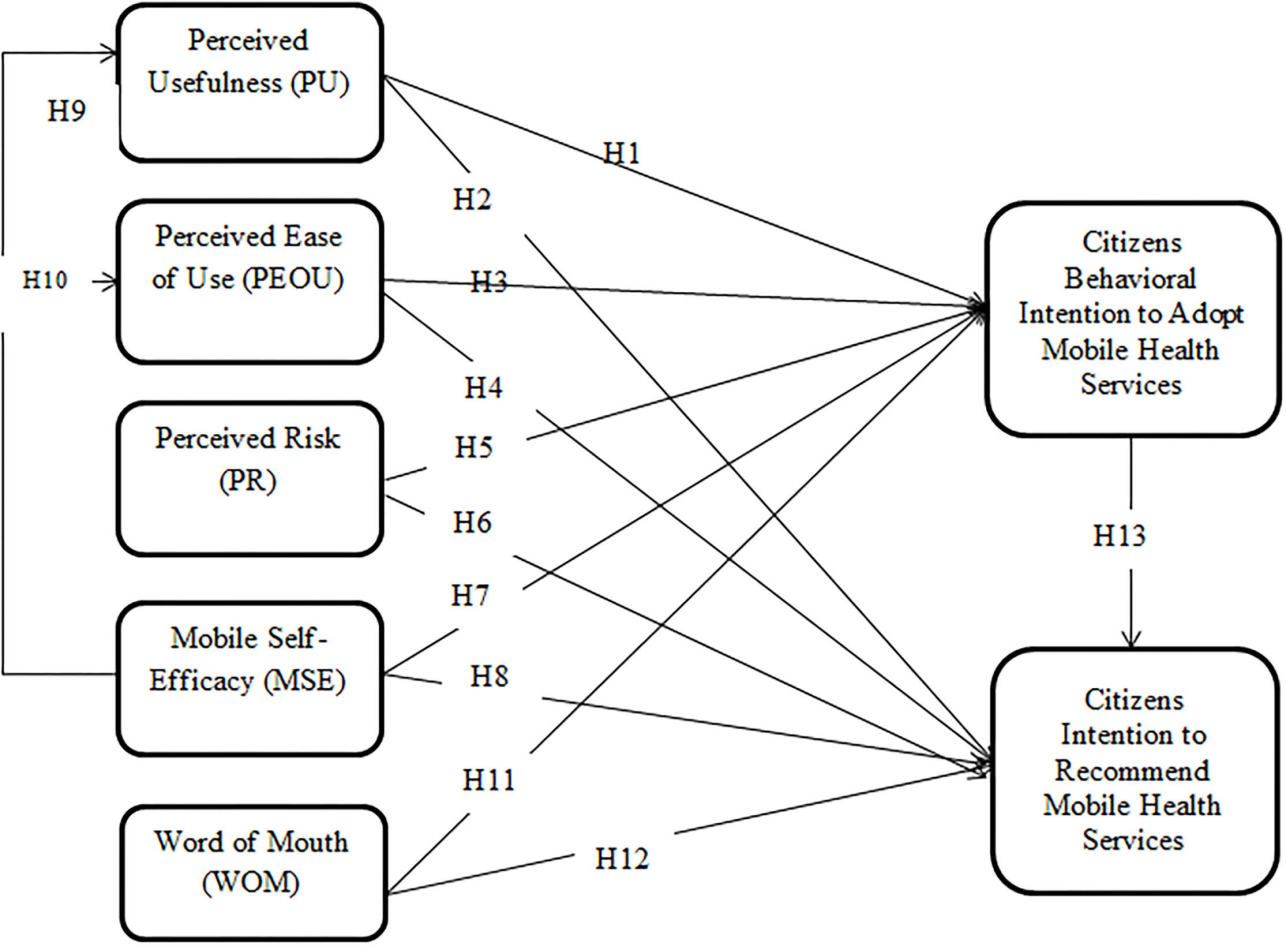
Research model.

The research model based on the hypotheses developed in the previous section is depicted in Figure 1. It illustrates a modified and extended TAM with constructs such as perceived risk, mobile self-efficacy, word of mouth, and recommendation behavior of mobile health services. TAM core constructs such as perceived usefulness and perceived ease of use, along with perceived risk, mobile self-efficacy, and word-of-mouth communication, are projected to impact both the intention to use behavior and recommendation behavior of mobile health services. Additionally, mobile self-efficacy is anticipated to drive both the perceived usefulness and perceived ease of use of mobile health services.

## Research Methodology

A questionnaire was developed based on a detailed literature review, and items were adapted from previous studies but were modified to fit the context of this study. The questionnaire was divided into two parts. The first section contained information about basic demographic information about the respondents, and the second part contained information about the variables explored in this study. The variables used were adopted as follows: perceived usefulness, perceived ease of use and behavioral intention to use (47, 48), intention to recommend (102), perceived risk (3, 103, 104), mobile self-efficacy (105, 106), and word of mouth (WOM) (99). The items were measured on a five- (5) point Likert scale, which ranged from strongly disagree (SD) to strongly agree (SA). The research items used in this study are attached as Appendix A.

Pre-testing and piloting of the survey were undertaken before it was administered; however, the results of the pre-testing and piloting were not included in the final data analysis. Pretesting and piloting are instrumental in soliciting feedback from potential respondents to gauge their understanding of the questions outlined in a survey and, thus, can contribute to improving the quality of research outcomes. The constructive feedback enabled revisions of some portions of the questionnaire. Pre-testing is a tool to make sure survey items and questions are properly articulated, and response options are relevant, comprehensive, and mutually exclusive, especially from the perspective of the respondents (107).

Copies of the questionnaire were administered (in English) to a cross-section of Ghanaian citizens through a social networking site (WhatsApp). Since the participants of this study were considered to be nonusers of MHS, on the first page of the research survey, an introduction that explained what MH is about was included in the survey. This was conducted to ensure that at least the participants had an idea of what m-health is and what it can do. WhatsApp, a social networking platform, was used as the appropriate means to reach the targeted population of this study, because it is among the most used social networking platforms in Ghana. The link was shared with multiple WhatsApp groups and through personal WhatsApp friends, and they were asked to forward and share it with their friends, family, and contact groups. The convenient sampling technique was used to research the respondents of the study, because convenience sampling is affordable and easy, and subjects are readily available. Convenient sampling is considered a form of non-probability sampling, which empowers researchers to select a sample based on easy reachability and accessibility for data collection (108, 109). The collection of data lasted for about 2 months (August and October 2021). After the administration process, a total of 645 valid responses were received. The gathered data were analyzed with SPSS and SmartPLS version 3 through the use of the structural equation modeling (SEM) technique. SEM is a technique that examines the extent to which a theoretical model is supported by data by testing of both latent and observed constructs (110). Besides, SEM makes it possible for researchers to undertake complex models with several variables, indicator constructs, and structural paths without putting distributional assumptions on data (111). Importantly, SEM encourages predictions in the estimation of statistical models, i.e., to offer statistical causal elucidations (111).

## Results And Data Analysis

### Demographic Profile

The demographic profiles of the respondents are shown in Table 4. There were more male respondents (55.3%) than female ones (44.7%). Most of the respondents were 41 years and above (25.1%), and the least age group was between the ages of 36 to 40 (11.5%). A large portion of them were undergraduate degree holders. In terms of occupation of the respondents, the majority were from the private sector (37.2%).

**Table 4:** Demographic profile of respondents.

**Item**	**Description**	**Frequency**	**Percentage**
Gender	Male	357	55.3
	Female	288	44.7
Age distribution	18–25	150	23.3
	26–30	158	24.5
	31–35	101	15.7
	36–40	74	11.5
	41+	162	25.1
Education level	Graduate(First Degree)	255	39.5
	Graduate(Masters)	148	22.9
	Graduate(PhD)	64	9.9
	Others	178	27.6
Occupation	Public Sector	128	19.8
	Private Sector	240	37.2
	Self-Employed	78	12.1
	Unemployed	129	20.0
	Student	70	10.9

### Measurement Model

Cronbach's alpha, composite reliability, average variance extracted (AVE), and factor loadings were used as quality standard to test the reliability and validity of the research constructs used in this study. The results of the measurement model are shown in Table 5. Factor loadings and Cronbach's alpha are recommended to have values above.7 (112, 113). The composite reliability should have a value of at least.8, and the average variance extracted should have values above.5 (113, 114). As indicated in Table 5, the required/recommended quality criterion for AVE, composite reliability, Cronbach's alpha, and factor loading are all met. This provides enough confidence to proceed to test the structural model of the study. Also, the discriminate validity of the variables was tested using the Fornell-Larcker criterion. The results of the discriminate validity are shown in Table 6. In the Fornell-Larcker criterion principle, a variable can be considered to have a discriminant validity if the square root of the average variance extracted (AVE) (in bold) is > the paired inter-correlation between latent variables (115). As illustrated in Table 6, all the diagonal variables are higher than their corresponding off-diagonal values. This, thus, confirms the discriminant validity of the constructs used.

**Table 5:** Quality criterion (AVE, composite reliability, alpha) and factor loadings.

**Construct**	**Item**	**Average variance extracted (AVE)**	**Composite reliability**	**Cronbach's alpha**	**Loadings**
Perceived Usefulness (PU)	PU1 PU2 PU3 PU4	0.886	0.968	0.956	0.982 0.853 0.970 0.954
Perceived Ease of Use (PEOU)	PEOU1 PEOU2 PEOU3 PEOU4	0.930	0.981	0.975	0.960 0.949 0.970 0.978
Perceived Risk (PR)	PR1 PR2 PR3	0.935	0.977	0.965	0.971 0.958 0.972
Mobile Self-Efficacy (MSE)	MSE1 MSE2 MSE3 MSE4	0.899	0.972	0.962	0.908 0.964 0.969 0.952
Word-of-Mouth (WOM)	WOM1 WOM2 WOM3	0.924	0.973	0.959	0.942 0.963 0.979
Behavioral Intention (BI)	BI1 BI2 BI3	0.892	0.961	0.939	0.974 0.912 0.947
Intention to Recommend (ITRC)	ITRC1 ITRC2 ITRC3	0.937	0.978	0.966	0.966 0.965 0.973

**Table 6:** Discriminant Validity.

**Items**	**PU**	**PEOU**	**PR**	**MSE**	**WOM**	**BI**	**ITRC**
PU	**0.941**						
PEOU	0.580	**0.964**					
PR	0.442	0.556	**0.967**				
MSE	0.523	0.691	0.662	**0.948**			
WOM	0.348	0.475	0.522	0.465	**0.961**		
BI	0.615	0.704	0.647	0.732	0.652	**0.944**	
ITRC	0.781	0.638	0.659	0.785	0.677	0.512	**0.968**

*Fornell-Larcker Criterion, Correlations of constructs and square roots of AVE (in bold). PU, Perceived Usefulness; PEOU, Perceived Ease of Use; PR, Perceived Risk; MSE, Mobile Self-Efficacy; WOM, Word-of-Mouth; BI, Behavioral Intention; ITRC, Intention to Recommend*.

### Structural Model

**Figure 2:**
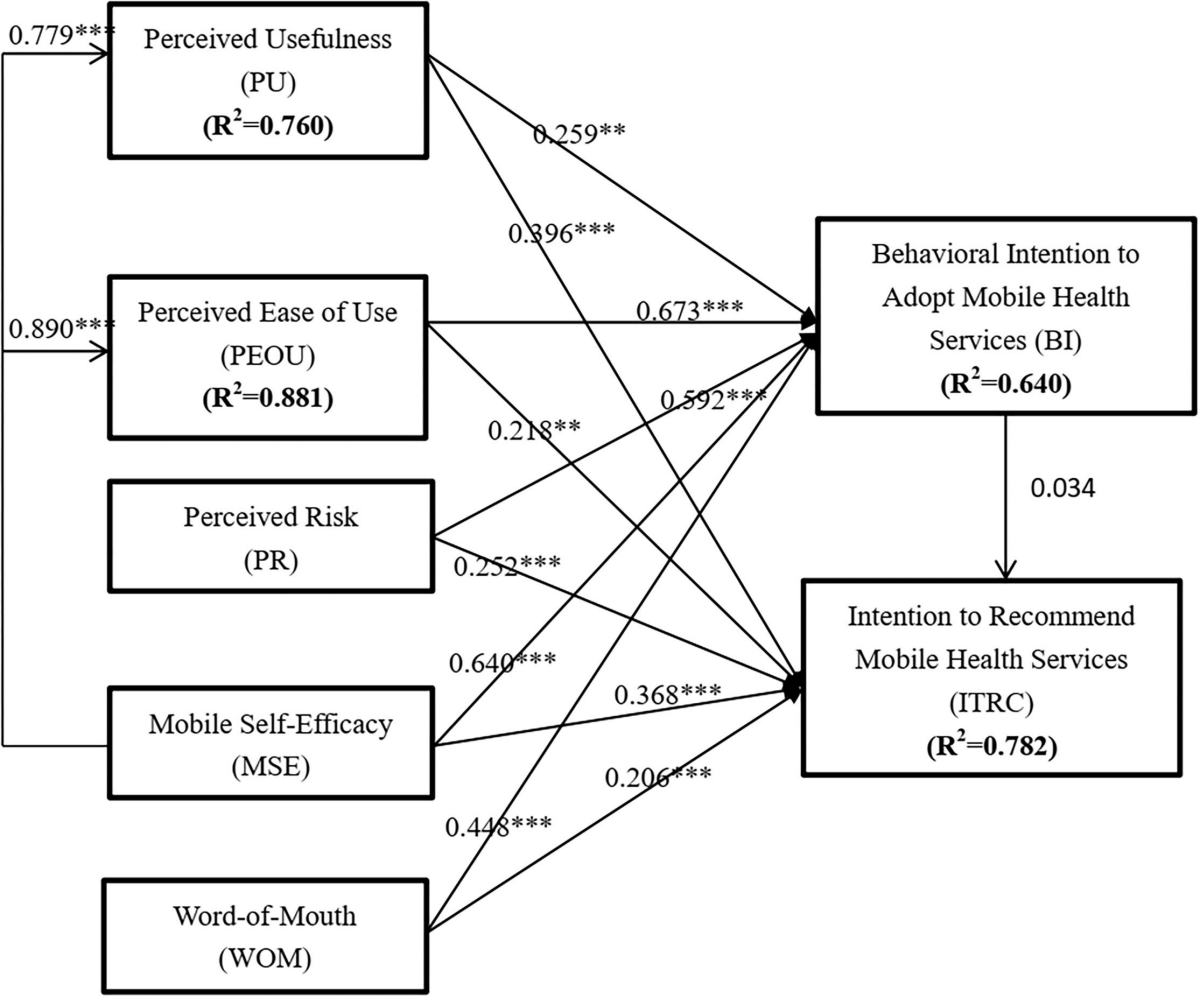
Validated structural model.

The results of the structural model tested are shown in Table 7 and graphically depicted in Figure 2. Perceived usefulness was significant in predicting both behavioral intention to use (β = 0259, *p* < 0.05) and intention to recommend the adoption of m-health services (β = 0.396, *p* < 0.05). Hence, H1 and H2 were supported. Perceived usefulness was also found to be a significant determinant of both behavioral intention to use (β = 0.673, *p* < 0.05) and intention to recommend the adoption of m-health services (β = 0.218, *p* < 0.05). H3 and H4 were also supported. In addition, perceived risk showed a significant but negative impact on both behavioral intention to use (β = 0.592, *p* < 0.05) and intention to recommend the adoption of m-health services (β = 0.252, *p* < 0.05). Thus, H5 and H6 were supported. Again, mobile self-efficacy showed a positive significant impact on both behavioral intention to use (β = 0.64, *p* < 0.05) and intention to recommend m-health services (β = 0.368, *p* < 0.05). Hence, H7 and H8 were statistically supported. Also, mobile self-efficacy was found to be a significant predictor of both perceived usefulness (β = 0.779, *p* < 0.05) and perceived use of m-health services (β = 0.89, *p* < 0.05). Therefore, H9 and H10 were supported. Furthermore, word of mouth (WOM) communication was found to be a significant determinant of both behavioral intention to use (β = 0.448, *p* < 0.05) and intention to recommend the adoption of m-health services (β = 0.206, *p* < 0.05). Accordingly, H11 and H12 were supported. Behavioral intention to use, however, was not significant in determining the behavioral intention to recommend the adoption of m-health services (β = 0.034, *p* >0.05).

**Table 7:** Hypotheses tested.

**Hypotheses**	**Path**	**β**	**T-value**	**Significance**	**Supported**
H1	PU → BI	0.259	2.272	0.024**	Yes
H2	PU → ITRC	0.396	6.909	0.000***	Yes
H3	PEOU → BI	0.673	6.074	0.000***	Yes
H4	PEOU → ITRC	0.218	2.565	0.011**	Yes
H5	PR → BI	0.592	10.002	0.000***	Yes
H6	PR → ITRC	0.252	5.806	0.000***	Yes
H7	MSE → BI	0.640	3.429	0.000***	Yes
H8	MSE → ITRC	0.368	4.082	0.000***	Yes
H9	MSE → PU	0.779	28.346	0.000***	Yes
H10	MSE → PEOU	0.890	21.473	0.000***	Yes
H11	WOM → BI	0.448	5.662	0.000***	Yes
H12	WOM → ITRC	0.206	4.627	0.000***	Yes
H13	BI → ITRC	0.034	0.840	0.402	No

(***p <0.01, ** p <0.05, *p <0.01). PU, Perceived Usefulness; PEOU, Perceived Ease of Use; PR, Perceived Risk; MSE, Mobile Self-Efficacy; WOM, Word-of-Mouth; BI, Behavioral Intention; ITRC, Intention to Recommend.

## Discussion

This study used the data collected from a section of Ghanaian citizens to investigate factors determining the adoption and recommendation intentions of m-health services in the Ghanaian context. The Technology Acceptance Model (TAM) was modified and extended with constructs such as perceived risk, mobile self-efficacy, word of mouth (WOM) communication, and intention to recommend m-health adoption. The results show that 12 of the 13 proposed hypothesized relationships are statistically supported. The results of the data analysis showed that perceived usefulness was found to be a significant predictor of both behavioral intention to use and recommend the adoption of m-health services. The significant impact of perceived usefulness on intention to use concurs with previous studies that showed that perceived usefulness is positively related to the intention to use m-health services (3, 87, 97, 116, 117). Besides, perceived ease of use of m-health services was a positive predictor of both behavioral intention to adopt and recommend the adoption of m-health services. Also, the impact of perceived ease of use on intention to use is in agreement with other studies that showed perceived ease of use of m-health services is a positive determinant of the intention to use m-health services (3, 26, 87, 118). Other scholars have also demonstrated that both the TAM/UTAUT key constructs of perceived usefulness (performance expectancy) and ease of use (effort expectancy) of mobile health systems drive the behavioral intention to use m-health (13, 119–121). The significant impact of both perceived usefulness and perceived ease of use on the citizens' intention to recommend the adoption of m-health services appears to be a special and key finding of this research. This is an indication that the benefits and ease of use associated with m-health services have the potential to encourage citizens to recommend the adoption of m-health services to others as well.

Furthermore, the test of the perceived risk construct showed that perceived risk was a negative but significant predictor of both intention to use and intention to recommend the adoption of m-health services. The negative but significant impact of perceived risk on the intention to use mobile health services agrees with the findings of other studies that reported that perceived risk is a significant determinant of the intention to use (3, 122, 123). This finding is a departure from studies that revealed perceived risk showed an insignificant impact on behavioral intention to use (124). The validated impact of perceived risk on recommendation intentions of m-health services seems to be a key finding of this study, since no study has, so far, experimented on this relationship. Also, the investigation of the mobile self-efficacy construct demonstrated that mobile self-efficacy was a significant determinant of both the behavioral intention to use and the intention to recommend the adoption of m-health services. The positive significant findings of mobile self-efficacy on the intention to use support findings that show that self-efficacy is a significant determinant of the intention to adopt (26, 97). Other research has further confirmed that the level of self-efficacy demonstrated toward the utilization of technology drives the intention to use such applications (124) but contradicts results indicating that self-efficacy does not influence the intention to use (125). Again, mobile self-efficacy was found to be significant in determining both perceived usefulness and perceived ease of use of m-health services. The findings on the significant impact of self-efficacy on both perceived usefulness and perceived ease of use of mobile health services corroborate findings that suggested self-efficacy is positively related to perceived usefulness and perceived ease of use (96).

Additionally, the analysis revealed that word-of-mouth (WOM) communication was a significant predictor of both the citizens' behavioral intention to adopt and recommend the adoption of m-health services. This empirical validation, again, appears to be an addition to the m-health adoption literature, since no study has tested this relationship. This means that the nature of the WOM communications from fellow citizens could influence both the intention of citizens to use and recommend to others to adopt m-health services. The importance of WOM has been highlighted to demonstrate that it influences the health behavior of individuals; as a result, the asymmetry of health information/knowledge is gradually improved because of people becoming more adequately informed (126, 127). Finally, the analysis interestingly showed that behavioral intention (BI) to use is not a significant predictor of the intention to recommend the adoption of m-health services. The possible explanation for the non-significant impact of BI on recommendation intention may be due to the limited diffusion of actual mobile health systems in Ghana and the fact that the respondents are non-users of m-health. This finding is a departure from another related study that showed that the behavioral intention to use the content of mobile payment (e-commerce) is positively related to the intention to recommend the adoption of new technologies (102). However, within the context of m-health services, this is among one of the few studies to have incorporated WOM communications on the adoption of m-health services as compared to studies such as (128, 129).

These findings have important practical and theoretical implications for the development and implementation of the m-health service delivery system for access to quality healthcare service delivery.

### Practical Implications

The first implication is that m-health services should be designed to provide greater benefits to improve citizens' access to better healthcare services than the traditional health delivery system. Also, the design of mobile health service application interfaces should be done with maximum attention to their ease of use potency. Attainment of the usefulness and ease of use associated with m-health services will encourage citizens to adopt them and recommend their adoption to other people in their immediate surroundings. Another implication is the impact that the perceived risk associated with m-health services can have on both intention to use and recommend. As best as possible, m-health practitioners, developers, and policymakers should design and implement an m-health system that reduces the perceived risk related to technology adoption applications such as m-health to consumers of m-health services. M-health systems, just like any other technology adoption application, should provide maximum protection to the personal and transactional information provided through the use of m-health services. Consumers of mobile health services should be assured that unauthorized persons or groups will not be granted access to their medical or services information or records without their consent. Also, putting in place proper regulatory laws can guide both m-health service providers and consumers on the engagement and interaction on m-health service platforms, and can provide adequate confidence to reduce the risks associated with this type of virtual mobile environment. These measures can reduce the perceived risk of consumers toward m-health services and, thus, will entice them to use and recommend the adoption of m-health services.

Although mobile phone technology has been with us for some time now, the issue of mobile self-efficacy continues to be an important consideration for its adoption. Hence, any mobile technology-related applications such as mobile health services must consider mobile self-efficacy for people to use mobile phones to access healthcare services. Mobile self-efficacy is vital to the success of mobile health service adoption, particularly in a developing country such as Ghana where access and use of smartphones in rural areas may be low or non-existent. Public health policymakers and developers can intensify education on mobile health usage, particularly in rural and remote areas, which will increase the belief in citizens' ability to operate or access healthcare through mobile phones (smartphones). This will provide the needed basic competencies, confidence, and knowledge to operate mobile health services, which will, in turn, drive them to use and recommend adoption to others as well. Besides, higher levels of mobile self-efficacy among citizens can enable them to appreciate the benefits and ease of use associated with the use of m-health services.

Finally, WOM communications about mobile health services can influence the citizens' adoption intentions and recommendations. This implies that opinions from friends, relatives, colleagues, and acquaintances about mobile health services can either have a positive effect or a negative effect on the adoption of m-health services. To avoid misconception or misinformation from close friends and relatives about m-health services in the form of WOM communications or person-to-person communications, m-health care service providers and relevant healthcare bodies should provide timely, quality, and relevant information about m-health services to citizens. The provision of adequate information about m-health services may positively influence the nature of WOM communications about m-health services, which will, in turn, have a direct positive impact on both citizens' behavioral intention to use and recommend the adoption of m-health services.

### Theoretical Implications and Contributions

The extension of the Technology Acceptance Model in the context of m-health adoption has some theoretical implications. First, perceived usefulness, perceived ease of use, perceived risk, mobile self-efficacy, and WOM communications are all significant predictors of both the behavioral intention to use and recommend the adoption of m-health services. Second, mobile self-efficacy is positively related to both perceived usefulness and perceived ease of use. Third, the behavioral intention to use is not significantly related to the citizens' intention to recommend. Also, perceived usefulness, perceived ease of use, perceived risk, mobile self-efficacy, and WOM jointly account for about 64 and 78.2%, respectively, of the variance in the behavioral intention to use and citizen's intention to recommend the adoption of m-health services. Mobile self-efficacy accounted for 76 and 88.1% of the factors determining, respectively, the perceived usefulness and ease of use of m-health services.

The major theoretical contributions of this study are that this is one of the first studies to have validated the significant impact of perceived usefulness, perceived ease of use, perceived risk, mobile self-efficacy, and WOM communications on the recommendation intentions of m-health adoption. The unique findings are different from studies that have also examined m-health adoption (130, 131). These contributions have provided empirical support for the extension of the Technology Acceptance Model (TAM) in the context of m-health adoption and, thus, are an important addition to the m-health adoption literature.

## Conclusion

Mobile health technology application in the healthcare sector is a new important innovation addition in the quest to provide quality public health services to citizens around the world, particularly in developing countries. Developing countries like Ghana can leverage mobile health applications to revitalize healthcare delivery systems/environments in areas of data collection, management, education and training, and promotion of health activities. Mobile collection of data can ensure faster access to data for decision-making and constant monitoring of patients' health conditions. Also, m-health systems, in terms of management revitalization, can contribute to the creation of health mobile information systems for workers' planning, control, and oversight. They improve administrative systems for managerial, financial, and supply chain management. Additionally, mobile-enabled health systems can be used for personal training with regular updates and reference materials. Health promotion aspects can ensure the delivery of messages *via* mobile devices directly to patients, supporting adherence to therapy and facilitating access to a medical service facility.

From the perspective of the sample investigated in this study, the behavioral intention to use and recommendation intentions are influenced by perceived usefulness, perceived ease of use, perceived risk, mobile self-efficacy, and WOM communications. Also, mobile self-efficacy is positive in determining the perceived usefulness and perceived ease of use of m-health services. These findings have provided empirical practical and theoretical implications for the development and deployment of m-health services from the Ghanaian perspective. Taking these validated factors into consideration will contribute greatly to the uptake of m-health services and, hence, its success as well. To adequately improve the development and diffusion of mobile health for sustainability in healthcare delivery in Ghana, the following are recommended and put forward for policymakers and government to increase the acceptability of m-health:

a. Continued deployment of ICT and mobile technology infrastructures especially in deprived regions to augment the development of m-health systems.
b. Implementation of an e/m-health policy framework to serve as a guide for healthcare institutions wishing to implement m-health systems.
c. Ensure adequate top management support for the use of mobile technology for the delivery of healthcare services.
d. Adequate security measures should accompany the design of m-health systems to secure the safety of users' information shared on m-health systems.
e. Undertake strong awareness campaigns, education, and training for patients, customers, and health professionals in the healthcare delivery ecosystem.
f. The healthcare system should be equipped with the required management and technical expertise to properly undertake mobile health treatment successfully.
g. Mobile health systems should be designed (usability) with ease-of-use features and flexible with long-term orientation for users to appreciate how a new technology can be beneficial to them.
h. Interoperability of m-health systems is needed to ensure compatibility for easy interaction of systems across technical and information platforms.
i. Develop sustainable funding strategies for large-scale implementation of m-health systems.
j. To achieve the cost-effectiveness of m-health development (interventions), m-health should be assessed in terms of cost, capacity needs, and resources against all other priorities.

## Limitations And Future Research

First, the sample size may not be representative; hence, the results should not be over-generalized. Second, this study used data collected from a cross-section of Ghanaians; thus, the results may not reflect the same outcome when applied in the context of another developing country. Another possible limitation is the issue of common method bias, since the data used were obtained from one single source and the general nature of the survey may not be reflective of the adoption of specific m-health technology systems. Fourth, the predictors examined in this study are not exhaustive of the factors determining the adoption of mobile health services in Ghanaian society. Hence, future studies should endeavor to explore other factors such as electronic WOM communications, resistance to change, demographic factors, m-health argument quality, health consciousness, and m-health source credibility on both the intention to use and recommend adoption of m-health services.

## Data Availability Statement

The original contributions presented in the study are included in the article/supplementary material, further inquiries can be directed to the corresponding author.

## Ethics Statement

Ethics review and approval/written informed consent was not required as per local legislation and institutional requirements.

## Author Contributions

The author confirms being the sole contributor of this work and has approved it for publication.

## Funding

This research was funded by the International Business Management (IBM) project (No. 203201100120) under the Postgraduate Curriculum System Construction Project of Jiangxi University of Science and Technology.

## Conflict of Interest

The author declares that the research was conducted in the absence of any commercial or financial relationships that could be construed as a potential conflict of interest.

## Publisher's Note

All claims expressed in this article are solely those of the authors and do not necessarily represent those of their affiliated organizations, or those of the publisher, the editors and the reviewers. Any product that may be evaluated in this article, or claim that may be made by its manufacturer, is not guaranteed or endorsed by the publisher.

## Appendix A: Research Items

### Perceived Usefulness (PU)

PU1: I will find m-health useful in my daily life.
PU2: I believe using m-health increases my chances of achieving things that are important to me.
PU3: I think using m-health increases my effectiveness in monitoring my health.
PU4: I believe using m-health increases my performance in monitoring my health.

### Perceived Ease of Use (PEOU)

PEOU1: Learning how to use m-health is easy for me.
PEOU2: My interaction with m-health is clear and understandable.
PEOU3: I find m-health easy to use.
PEOU4: It is easy for me to become skillful at using m-health.

### Perceived Risk (PR)

PR1: I think it is risky to provide my personal information in the m-health system.
PR2: I think it is risky to input my bank card information for registration, bill paying, or prepaid.
PR3: Entering personal information over the m-health system is unsafe.

### Mobile Self-Efficacy (MSE)

MSE1: I believe I can use the m-health if there was no one around to tell me what to do.
MSE2: I believe I can use m-health even if I have never used similar technology before.
MSE3: I am confident that I can effectively check my health status using m-health.
MSE4: I think do not need any special training skills to be able to use m-health.

### Word-of-Mouth (WOM)

WOM1: Exactly I will tell the other person that m-health is very good.
WOM2: I am proud to tell others that I use m-health service.
WOM3: I will share this good service of m-health with others quite frequently.

### Behavioral Intention (BI)

BI1: I am determined to use mobile health to monitor my health in my daily life.
BI2: I intend to use mobile health to monitor my health.
BI3: I foresee using mobile health to monitor my health in the future.

### Intention to Recommend (ITRC)

ITRC1: I find it wise to suggest to my family and friends to use an m-Health to monitor their health.
ITRC2: I think based on my positive interaction with m-health; I will recommend its use to others to monitor their health.
ITRC3: I think I will always recommend the use of m-Health system to monitor people's health.
